# Fixed muscle synergies and their potential to improve the intuitive control of myoelectric assistive technology for upper extremities

**DOI:** 10.1186/s12984-018-0469-5

**Published:** 2019-01-07

**Authors:** Tim A. Valk, Leonora J. Mouton, Egbert Otten, Raoul M. Bongers

**Affiliations:** Center for Human Movement Sciences, University of Groningen, University Medical Center Groningen, Antonius Deusinglaan 1, 9713 AV Groningen, the Netherlands

**Keywords:** Assistive technology, Muscle synergies, Point-to-point movements, Upper extremity, Electromyography, Intuitive control

## Abstract

**Background:**

Users of myoelectric controlled assistive technology (AT) for upper extremities experience difficulties in controlling this technology in daily life, partly because the control is non-intuitive. Making the control of myoelectric AT intuitive may resolve the experienced difficulties. The present paper was inspired by the suggestion that intuitive control may be achieved if the control of myoelectric AT is based on neuromotor control principles. A significant approach within neurocomputational motor control suggests that myosignals are produced via a limited number of fixed muscle synergies. To effectively employ this approach in myoelectric AT, it is required that a limited number of muscle synergies is systematically exploited, also when muscles are used differently as required in controlling myoelectric AT. Therefore, the present study examined the systematic exploitation of muscle synergies when muscles were used differently to complete point-to-point movements with and without a rod.

**Methods:**

Healthy participants made multidirectional point-to-point movements with different end-effectors, i.e. with the index finger and with rods of different lengths. Myosignals were collected from 22 muscles in the arm, trunk, and back, and subsequently partitioned into muscle synergies per end-effector and for a pooled dataset including all end-effectors. The exploitation of these muscle synergies was assessed by evaluating the similarity of structure and explanatory ability of myosignals of per end-effector muscle synergies and the contribution of pooled muscle synergies across end-effectors.

**Results:**

Per end-effector, 3–5 muscle synergies could explain 73.8–81.1% of myosignal variation, whereas 6–8 muscle synergies from the pooled dataset also captured this amount of myosignal variation. Subsequent analyses showed that gradually different muscle synergies—extracted from separate end-effectors—were exploited across end-effectors. In line with this result, the order of contribution of muscle synergies extracted from the pooled dataset gradually reversed across end-effectors.

**Conclusion:**

A limited number of muscle synergies was systematically exploited in the examined set of movements, indicating a potential for the fixed muscle synergy approach to improve the intuitive control of myoelectric AT. Given the gradual change in muscle synergy exploitation across end-effectors, future research should examine whether this potential can be extended to a larger range of movements and tasks.

## Background

Within current rehabilitation practice, a substantial focus is on applying assistive technology (AT) to help patients with neuromotor deficits regain functionality in their daily activities. Often, myosignals are used to control the AT, e.g. in myoelectric prostheses [1–3], myo-powered electric wheelchairs [4–6], and movement supporting devices, such as exoskeletons [7–9] and orthoses [10, 11]. Furthermore, myosignals have been applied in other human-machine interfaces which are relevant for the independence of patients with neuromotor deficits, such as in the control of a personal computer [12] or the teleoperation of robotic arms [13–16]. However, despite the technological advancement in many devices, patients often still experience problems to control myoelectric AT in daily life [17–21]. For instance, movements with myoelectric AT are non-smooth and require high levels of attention [18, 22]. One of the aspects that could cause these problems is that the control of myoelectric AT is non-intuitive, as muscles have a different function during the control of actions with myoelectric AT compared to the same action in the non-affected situation (cf. [23, 24]). For example, in hand prosthetics, the action of closing and opening of the hand is controlled with remaining parts of wrist or elbow flexors and extensor muscles—depending on the level of amputation. This function is arguably different from the original function of these muscles, i.e. flexing and extending the wrist or elbow, respectively. The present paper was inspired by the suggestion that an intuitive interface between user and device would aid the effective control of myoelectric AT (cf. [25–27]). Furthermore, the present paper was grounded on the idea that this intuitive control can be achieved if the design of myoelectric AT is based on knowledge of the neuromotor control principles underlying the production of myosignals (cf. [28–31]). Therefore, we explored the extent to which the fixed muscle synergy approach [32–37], a proposed control principle from the field of neurocomputational motor control, could form a basis to improve the intuitive control of myoelectric AT for upper extremities.

The basics of the fixed muscle synergy approach are that myosignals are produced by simultaneously activating a limited number of fixed muscle groups consisting of functionally related muscles across multiple joints, i.e. the muscle synergies. These fixed muscle synergies are proposed to be interneuronal networks, which do not change over time, and are organized at the level of the spinal cord (cf. [32, 33, 35, 36]). Activation of one of these networks leads to proportional activation of the muscles within a synergy. In that way, fixed muscle synergies serve as primitives, which—if conjointly activated in a time-varying way—can produce appropriate myosignals moving the limb across different tasks and conditions [32–37]. Over the last decade, findings of muscle synergies in myosignals in a variety of human behaviors—such as reaching in three dimensions [38–44], upper extremity visuomotor adaptation [45], force control [46], planar reaching [47–49], upper extremity movements in virtual reality [50], walking [51–53], and postural control [54–56]—have been offered as evidence for the existence of fixed muscle synergies as used neuromotor control principle. The relevant question for the present paper is whether the proposed principle of fixed muscle synergies could aid in controlling myoelectric AT in an intuitive way.

The successful improvement of the intuitive control of myoelectric AT with fixed muscles synergies requires that muscles are indeed organized into fixed synergies and that these synergies are systematically exploited during the different use of muscles as required in the control of myoelectric AT. The aim of the present study was to gain new insights in the viability of the fixed muscle synergy approach for its application in upper extremity assistive devices. Therefore, the present study assessed whether a limited number of muscle synergies was systematically exploited when the same task had to be produced while muscles were used differently. In the present experiment, able-bodied participants made multidirectional point-to-point movements with end-effectors of different lengths—i.e. their index finger and with a rod of varying length. Due to the introduction of end-effectors of different lengths, the same point-to-point movement with the tip of the end-effector had to be produced while postural angles, and thus the use of muscles, varied over a large range [57–59]. Explaining this behavior in terms of fixed muscle synergies, the same set of fixed muscle synergies had to be activated with different time-varying signals to produce the appropriate myosignals moving the tip of the end-effector both with and without the use of rods. The potential finding that a limited number of muscle synergies is systematically exploited to produce myosignals when muscles are used differently across end-effectors would encourage the use of the fixed muscle synergy approach to improve the intuitive control of myoelectric AT for upper extremities. Alternatively, the set of muscle synergies might substantially differ across end-effectors. Such a finding would warrant further examination of the idea of fixed muscle synergies as neuromotor control principle underlying the production of myosignals and places limitations on its potential to improve the intuitive control of myoelectric AT for upper extremities.

## Methods

### Participants

Eleven right-handed participants (mean age 23.9 ± 2.5 years, five males) took part in the experiment. Participants had no neuromotor deficits and all had normal or corrected-to-normal sight. Participants received verbal and written information about the procedures and signed an informed consent before the start of the experiment.

### Experimental set-up

**Fig. 1:**
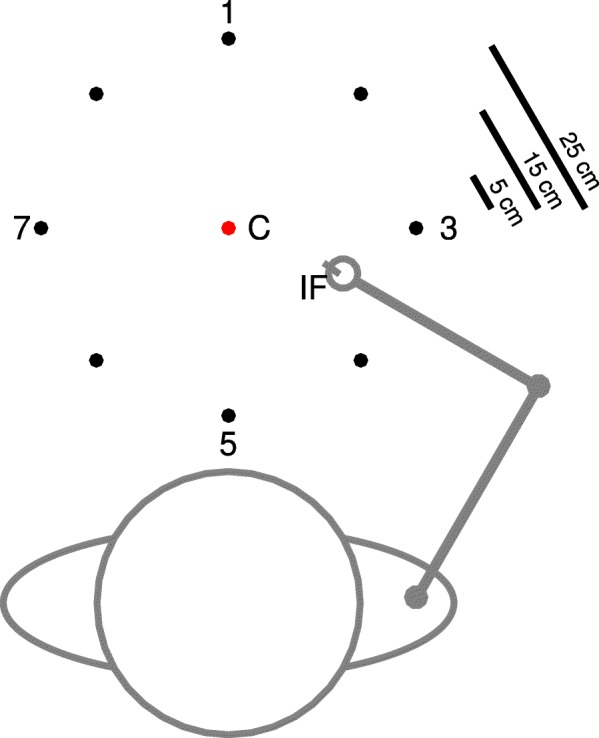
Experimental set-up, as seen from above

In the experiment, participants made point-to-point movements with the tip of their index finger or the tip of a rod that was attached to the index finger. During the point-to-point movements, eight peripheral targets that were distributed equally around a center point (Fig. 1) were tested. All targets had a diameter of 1 cm, and the distance between the center point and targets was 25 cm. The center point and targets were printed on a piece of paper (A2 size, landscape orientation), which was presented on a table in front of the participants. The rods used during the experiment were made of aluminum; had a diameter of 0.5 cm; a length of 5, 15, or 25 cm; and a mass of 4, 12, or 20 g; respectively. These rods were attached to an aluminum holder (weight: 50 g) that was attached to the dorsal side of the index finger. Furthermore, at the ventral side of the index finger, a small aluminum plate was attached to prevent movement of the interphalangeal joints while allowing free motion of the metacarpophalangeal joint (cf. [59]).

Five rigid bodies, triangular in shape, containing one infrared light-emitting diode (LED) in each of the three corners, were attached to the right side of the participants’ body, following Van Andel et al. [60]. The rigid bodies were placed on the sternum, the flat part of the acromion, laterally to the upper arm just below the insertion of the deltoid, dorsally to the lower arm just proximal to the ulnar and radial styloids, and to the dorsal surface of the hand. The rigid bodies attached to the sternum and the upper arm had a leg length of 6 cm; the other three rigid bodies had a leg length of 4 cm. Another set of three LEDs was attached to the aluminum holder on the index finger. Positional data for the eighteen LEDs was gathered with an Optotrak 3020 system (Northern Digital, Waterloo, Ontario, Canada), using two synchronized units that sampled at 100 Hz. To relate positions of the LEDs in space to the anatomy of the participants and the movement of the rods, nineteen bony landmarks of the participants and the tips of the rods were digitized using a pointer device [60].

Myosignals of 22 muscles (flexor carpi radialis [FCR]; flexor carpi ulnaris [FCU]; extensor carpi ulnaris [ECU]; extensor carpi radialis [ECR]; pronator teres [PRO]; brachioradialis [BRO]; brachialis [BRA]; long and short head of the biceps brachii [BIL and BIS, respectively]; long, medial and lateral head of the triceps brachii [TRLo, TRMe, and TRLa, respectively]; pectoralis major [PEC]; anterior, middle and posterior part of the deltoid [DEA, DEM, and DEP, respectively]; superior, medial, and inferior part of the trapezius [TRS, TRM, and TRI, respectively]; infraspinatus [INF]; teres major [TER]; and latissimus dorsi [LAT]) were recorded using surface electromyography (EMG) as measured by two Porti systems (TMSi, Enschede, the Netherlands), which were synchronized with the Optotrak system. One Porti device sampled in millivolts with no amplification at a frequency of 2048 Hz, whereas the other device sampled in millivolts with twenty times amplification at a frequency of 1600 Hz. In the data analysis, raw data from both systems were converted to signals in millivolts, with no amplification. Before electrode attachment, hair was removed from the attachment location if necessary, and the skin was cleaned using denatured alcohol. After electrode placement, the correctness of placement was determined by having the participant perform a number of movements in which the targeted muscle was involved [61], while visually checking whether EMG activity matched expectations. Corrective placements were performed if necessary.

After positional LEDs and EMG electrodes were attached, participants were gently strapped against the extended back of a chair, in such a way that trunk motion during the point-to-point movements was prohibited while the motion of the shoulder was free. Furthermore, an elbow placer was positioned next to the participant to standardize the participants’ position of the olecranon at the start of each trial, which ensured a similar start posture when the tip of the end-effector was held at the center point.

### Experimental procedure

Point-to-point movements were made from the center point to one of eight peripheral targets (center-out movement) and back from this peripheral target to the center point (out-center movement). Before each trial, the experimenter indicated to the participant towards which target had to be pointed. Subsequently, the experimenter gave a start signal, after which participants reached as quickly and accurately as possible from their start point to the selected target. After each center-out trial, the next movement to be made was its out-center counterpart back to the center point. Note that the instruction was to move as quickly as possible but that the response time was not emphasized. After each movement, participants were required to hold the tip of the end-effector at the terminal position for a maximum of 1 s.

### Design

In the present study, the participants performed 320 trials. Each couple of center-out and out-center movements involving one of the eight peripheral targets was repeated five times in a random order as a block for each of the four end-effectors (index finger and the three rods). These blocks were also presented in random order. After these 320 trials, participants performed another 240 trials. These trials were not used in the present study.

### Data analysis

#### End-effector and joint-angle kinematics

Using rigid body transformations, the movement trajectory of the tip of the end-effector was computed from the Optotrak LEDs at the aluminum holder. Using this movement trajectory of the end-effector tip, the start of each movement in a trial was determined as the last frame in the data before the tangential velocity—computed as the square root of summed squares of derivatives of the 3D positional data—of the end-effector tip went above a speed of 25 mm/s. In a similar way, the end of each movement in a trial was determined as the first frame in the data after the tangential velocity of the end-effector tip dropped below a speed of 25 mm/s, with the additional requirement that the tip of the end-effector had to be within a radius of 10 mm around its final position. The time between start and end of the movement was defined as movement time. To determine the accuracy at the target, the absolute error, defined as the absolute difference between the position of the tip of the end-effector and the center of the target at movement termination, was computed.

To describe the executed movements in terms of joint-angles, three joint-angles that contributed most to the movement (cf. [59])—i.e. shoulder plane of elevation, shoulder inward-outward rotation, and elbow flexion-extension—were computed using ISB guidelines for the upper extremity [62]. These joint-angles were derived from segment orientations using the digitized bony landmarks and the Optotrak LEDs attached to the rigid bodies. Joint-angle trajectories were normalized over time.

#### EMG processing and muscle synergy extraction

The raw EMG signals for each trial were band-pass filtered based on SEMIAN guidelines (4th order Butterworth filter, 20–500 Hz), rectified, and low-pass filtered (4th order Butterworth filter, 10 Hz) to determine the linear envelope of the EMG signal. Before performing further analyses, all EMG signals were checked for artifacts, and divergent signals—e.g. signals with extremely high amplitudes—were removed from the analysis. For the approved linear envelopes, a linear ramp—determined by calculating a line from the average activation from trial onset until 200 ms before movement onset to the average activation from 200 ms after movement termination until trial termination—was subtracted from these signals to exclude muscle activity due to posture and work against gravity (cf. [38]). Subsequently, the portion of the EMG linear envelope from 200 ms before movement onset to 200 ms after movement termination was selected for further analysis. This portion of the signal was resampled to 100 data points using a cubic spline, and per muscle, the EMG signals were normalized in amplitude to the highest value that that muscle exerted in the whole experiment. Last, these normalized signals were averaged across trials from every movement direction (center-out or out-center) and target combination within every end-effector.

In the fixed muscle synergy approach, it is assumed that a set of S myosignals, in this study 22, over time T can be obtained by combining a limited number of N muscle synergies W, representing the proportional activation level of each muscle in the synergy (in an S times N matrix), with a time-varying activation signals C (in an N times T matrix), according to the following equation:

1 $$ M=W\ast C+e $$

in which *e* represents the error between the observed myosignals M and the modeled myosignals W*C.

A non-negative matrix factorization algorithm [63] was used to extract the muscle synergies W and time-varying activation signals C from the observed myosignals in matrix M. Per participant, this matrix M was composed five times: four times separately for every end-effector including all processed and averaged myosignals from every movement direction and target combination within that end-effector, and one time as a pooled dataset including all processed and averaged myosignals from all end-effectors.

**Fig. 2:**
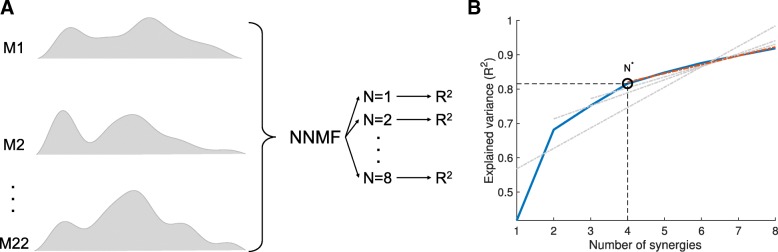
Graphical representation of muscle synergy extraction process. **a** Processed myosignals of 22 muscles (M1 to M22 in the figure) were factorized into muscle synergies with a non-negative matrix factorization algorithm, for a range of 1–8 possible number of muscle synergies N. For each N in this range, the amount of variance in myosignals that these N muscle synergies could explain (R^2^) was determined using eq. 2. **b** Explained variances R^2^ and possible number of muscle synergies N plotted in an R^2^ versus N curve (blue curve in the figure). By repeatedly fitting straight lines through decreasing portions of the R^2^ versus N curve (grey dotted lines in the figure), it was determined which portion of the curve was essentially straight (red dotted line represents fitted line through this essentially straight part of the curve). The starting point of this essentially straight portion of the curve was established as the to-be-found number of muscle synergies N^*^ (in the present example 4)

For the extraction of muscle synergies from the myosignals of every separate end-effector, the algorithm was executed 25 times for a range of 1–8 possible number of muscle synergies N (cf. [38]) (Fig. 2a). To increase the possibility of finding a global minimum error between observation and model, different initial matrices W and C were taken for every iteration. For every possible number of muscle synergies N, the set of muscle synergies extracted from these 25 iterations with the highest explained variance (R^2^) of the observed myosignals M was retained. This R^2^ was computed as follows:

2 $$ {R}^2=1-\frac{SSE}{SST}. $$

In this eq. 2, SSE is the sum of squared errors of the data reconstructed by the muscle synergies, and SST is the sum of squared residuals of the data with respect to the mean of the different rows of the matrix M.

Subsequently, for every end-effector, the to-be-found number of muscle synergies N^*^ within an end-effector was selected based on the following rationale [38], (cf. [64]). If N^*^ is the to-be-found number of muscle synergies, it is expected that a change in the slope of the R^2^ versus N curve from curved to straight appears at the number of N = N^*^ muscle synergy combinations. This change is expected because the additional muscle synergies will only represent variation which is attributable to noise, and hence will explain similar portions of data variation. To objectively find this spot in the curve, a linear regression procedure was used to examine for which N the R^2^ versus N curve was essentially straight [64]. This spot was found by fitting straight lines through decreasing portions of the R^2^ versus N curve, removing the smallest N of the portion of curve per iteration (Fig. 2b). The first N for which the mean square residual error of regression line N to N^max^ with the R^2^ curve was < 10^− 4^ (cf. [38]) was selected as the number of muscle synergies in that specific end-effector.

Also for the pooled dataset containing the myosignals from all end-effectors, the non-negative matrix factorization algorithm was run 25 times, but now for a range of 1-N^PooledMax^ possible total number of muscle synergies. In this, N^PooledMax^ is the sum of the number of muscle synergies as estimated in the end-effectors separately. From these N^PooledMax^ different total muscle synergies sets, the total muscle synergy set for the pooled matrix was determined as the minimal number of muscle synergies that could equally well describe the myosignals from every end-effector as the muscle synergies separately extracted from these end-effectors could.

#### Assessment of the exploitation of muscle synergies

Three strategies were used to assess the exploitation of muscle synergies across end-effectors. First, the similarity of the structure of individual muscle synergies across end-effectors was assessed by i) visually perusing the muscle synergies ii) determining the normalized dot product (NDP) of two muscle synergies stemming from two different end-effectors, and iii) examining whether clusters of similar muscle synergies exploited across all end-effectors could be formed. Second, the explanatory ability of muscle synergies across end-effectors was assessed by i) determining, via the cosine of principal angles (CPA), whether two total muscle synergy sets extracted from two separate end-effectors could span the same subspace in the myosignal space, and ii) examining whether myosignals from one end-effector could be reconstructed with the total muscle synergy set from another end-effector. Third, the contribution of muscle synergies as extracted from the pooled dataset was evaluated across end-effectors.

##### Comparison of the structure of individual muscle synergies extracted from separate end-effectors

To obtain a first impression of the similarity of structure of individual muscle synergies across end-effectors, the proportional activation levels between muscles within individual muscle synergies were visually compared across these end-effectors. After this visual comparison, the similarity in structure of individual muscle synergies across end-effectors was determined mathematically with the NDP—calculated as the fraction of the scalar product of two muscle synergies and the product of their norms [48, 64–66]. This calculation was done in an end-effector-pair-wise way, in which all individual muscle synergies from one end-effector were compared with all individual muscle synergies from another end-effector on basis of their structure. From these comparisons, best matching muscle synergies, as indicated by their NDP, were paired, and the NDPs belonging to these matched muscle synergies were ranked in a descending order. This NDP analysis showed in which couple of paired muscle synergies similarity dropped below threshold—in the present study 0.9, (cf. [48, 64, 65])—which is indicative for the number of individual muscle synergies that showed similarity in their structure between two end-effectors.

To assess across which end-effectors a certain muscle synergy was exploited, individual muscle synergies that had a similar structure across end-effectors were clustered into groups using k-means clustering [67–69]. Over different iterations, k groups of clustered muscle synergies were formed—in which for each iteration, k increased by 1. This iterative process was repeated until all clusters contained only muscle synergies that had a similar structure. This similarity was assessed by comparing all muscle synergies within a cluster in a pair-wise manner using the NDP. The clustering process was ended if all clusters of muscle synergies exhibited a mean NDP—as averaged across the NDPs of all possible pairs within a cluster—that was larger than 0.9. From the characteristics of these clusters—i.e. the number and origin of the muscle synergies per cluster—we could deduce which muscle synergies were exploited across end-effectors.

##### Comparison of the explanatory ability of total muscle synergy sets extracted from separate end-effectors

In principle, it is mathematically possible that two total muscle synergy sets that differ in structure can explain the same assemblage of myosignals, and thus span the same subspace in myosignal space. In other words, it is possible that for the same subspace in myosignal space two total muscle synergy sets which differ in structure are extracted. Thus, even if the myosignals as observed in two end-effectors belong to the same subspace, it is possible that muscle synergies with different structures are extracted for these two end-effectors. In that case, the dissimilarity in the structure of individual muscle synergies, as assessed with the analyses presented above, stems from other factors, e.g. computational chance or noise, than a behavioral phenomenon. Therefore, we controlled for this possibility by assessing whether total muscle synergy sets—as extracted from the different end-effectors—had the same explanatory ability, i.e. could explain the same assemblage of myosignals. As a first assessment, we compared the similarity of two subspaces spanned by two total muscle synergy sets—as extracted from two end-effectors—by calculating the CPA between the dimensions of these two subspaces [48], ([70] p. 603–604). Similar to the comparison of NDPs, the comparison of subspaces was done in an end-effector-pair-wise manner, in which CPAs had to exceed the threshold of 0.9 [48, 64, 65] to be classified as similar. As with the NDP, the calculated CPAs were ranked in a descending order, such that this CPA analysis showed for which dimension the CPA dropped below threshold, indicative for the number of dimensions that were shared across end-effectors.

The second assessment of the explanatory ability of total muscle synergy sets examined whether the total muscle synergy set from one end-effector could explain myosignals from another end-effector [47, 48]. The myosignals from every end-effector were reconstructed with the total muscle synergy set from another end-effector using a linear least squares method with a non-negativity constraint. This method determined the time-varying activation coefficients C with respect to this total muscle synergy set W. The quality of reconstruction was determined by computing the R^2^ of this reconstruction, computed using eq. 2 presented above. Per reconstruction, the algorithm was run 25 times, with different initial coefficients for C, and the solution with the highest R^2^ was retained for analysis.

##### Evaluation of the contribution of muscle synergies extracted from the pooled dataset

Last, the contribution to the explanation of myosignals across end-effectors of the muscle synergies as extracted from the pooled dataset was examined. The contribution for each of these muscle synergies was determined by assessing the change in explained variance of the reconstruction of the myosignals of every end-effector when a muscle synergy was removed from the total set extracted from the pooled dataset. The reconstruction of myosignals with this total set minus one muscle synergy was determined using the same linear least squares method with a non-negativity constraint as presented above. As with other factorization procedures presented above, the algorithm was run 25 times, and the solution with the highest R^2^ was retained to compute the change in R^2^ with respect to the R^2^ of the total muscle synergy set. This procedure was repeated for every muscle synergy in the total set. Based on the change in explained variance, muscle synergies were ordered from most to least contributing for every end-effector.

### Statistical analysis

Potential differences in the number of muscle synergies across end-effectors were examined using the Friedman’s test. Bootstrap statistics, with a resampling of 10,000 times, were used to determine the 95% confidence interval around the sample means for NDP and CPA values across participants. These confidence intervals were used to check whether NDP and CPA sample means statistically differed from the selected threshold of 0.9, indicated by the whole confidence interval laying below threshold. To check for differences in R^2^ of myosignals reconstructed with a set of muscle synergies extracted from different end-effectors, one-way repeated measure ANOVAs, with end-effector (index finger, 5 cm, 15 cm, and 25 cm) as a within-subject factor, were used. Before entering the ANOVA, variables were checked both visually and with the Shiparo-Wilk test on their normality. No deviations from normality were found. If within these ANOVAs the assumption of sphericity was violated, the Greenhouse-Geisser correction was used. Furthermore, significant effects within these ANOVAs were further examined using post-hoc Bonferroni corrected pair-wise t-tests. ANOVA effects were interpreted with the generalized eta-squared (*η*^*2*^_*G*_) [71, 72], which was interpreted with 0.02 as a small effect, an effect size of 0.13 as a medium effect, and an effect size of 0.26 as a large effect [72], ([73] p. 413–414). Last, per participant, as well as for all participants together, obtained orders of muscle synergy contribution—for the muscle synergies extracted from the pooled dataset—were compared on their similarity in an end-effector-pair-wise way based on Kendall’s tau coefficient. Kendall’s tau coefficients were tested for significant deviation from zero. For all statistical analyses, an alpha level of 0.05 was taken as a threshold for statistical significance. All statistical analyses were performed using SPSS version 22 and Matlab version R2016a.

## Results

### Note on included participant data

Due to technical issues regarding the acquisition of bony landmarks of two participants (1 and 4)—needed for a positional description of their arm movements—we could not determine the joint-angle trajectories of these two participants. Fortunately, for these participants, the digitization of the various end-effector tips was successful. Therefore, also for these two participants, we could gather the movement trajectory of the tip of the end-effector using rigid body transformations, which was vital for the selection of the part of the recorded myosignals that was taken for further analyses. Thus, the results on joint-angle kinematics are based on data of only nine out of eleven participants; the rest of the results are based on data of all eleven participants.

### End-effector and joint-angle kinematics

**Fig. 3:**
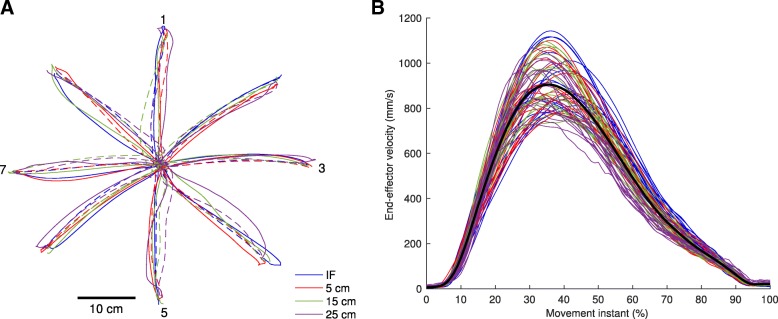
End-effector kinematics of performed point-to-point movements with different end-effectors. **a** Example of end-effector trajectories of one representative participant towards (solid lines) and from (dashed lines) the different targets for the different end-effectors. **b** End-effector velocity profiles for every participant, as averaged across all trials for every target and movement direction combination for every end-effector (indicated by the different colors, for legend see Fig. 3a). Black line denotes average velocity profile across all participants, all end-effectors, and all target and movement direction combinations

**Fig. 4:**
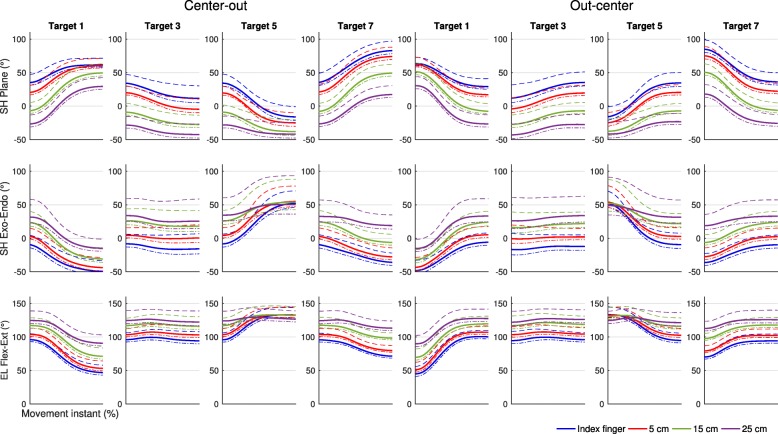
Joint-angle trajectories for a selection of point-to-point movements as performed in the experiment. Each panel represents the mean joint-angle trajectories as averaged across participants, as performed in point-to-point movements with the different end-effectors towards and from a selection of the different targets that occurred in the experiment. Dashed lines represent the standard deviation, dotted lines the standard error of the mean

Participants completed the task with a high accuracy in every point-to-point movement (average error of 6.6 ± 3.6 mm), and with an average movement time of 0.65 ± 0.16 s (IF: 0.59 ± 0.14 s; 5 cm rod: 0.63 ± 0.17 s; 15 cm rod 0.66 ± 0.16 s; 25 cm rod 0.73 ± 0.16 s). Examination of average movement times per participant showed that the tendency of longer lasting movements with longer end-effectors was present in all participants (Table 1). Trajectories of the tip of the end-effector were slightly curved (see for representative example Fig. 3a), exhibited bell-shaped velocity profiles (Fig. 3b), and showed small differences for the different end-effectors. Furthermore, movements in the three examined joint-angles were smooth, and, not unexpected, the joint-angle trajectories depended on the direction in which the movement was produced (Fig. 4). Also, joint-angle trajectories differed when different end-effectors (i.e. index finger or rod) were used to produce the point-to-point movements (Fig. 4), yet the shape of joint-angle trajectory was, most of the times, similar across end-effectors. For instance, in point-to-point movements towards target 5, participants always used a similar shape of the extension trajectory in the elbow to reach the target (Fig. 4, third row, first column panel). Such similarities could also be found in other joint-angle and target combinations.

**Table 1:** Average (± standard deviation) movement times (s) per participant, as averaged across all movements within an end-effector and across all movements in the whole experiment

Participant	Index finger	5 cm rod	15 cm rod	25 cm rod	Experiment
1	0.64 ± 0.13	0.65 ± 0.13	0.68 ± 0.12	0.75 ± 0.16	0.68 ± 0.14
2	0.80 ± 0.15	0.88 ± 0.17	0.81 ± 0.16	0.87 ± 0.15	0.84 ± 0.16
3	0.55 ± 0.10	0.60 ± 0.10	0.62 ± 0.13	0.73 ± 0.15	0.62 ± 0.14
4	0.49 ± 0.10	0.54 ± 0.10	0.64 ± 0.10	0.65 ± 0.13	0.58 ± 0.13
5	0.52 ± 0.08	0.54 ± 0.08	0.55 ± 0.10	0.70 ± 0.13	0.58 ± 0.12
6	0.63 ± 0.11	0.72 ± 0.14	0.77 ± 0.16	0.81 ± 0.16	0.73 ± 0.16
7	0.55 ± 0.10	0.60 ± 0.10	0.60 ± 0.09	0.66 ± 0.14	0.60 ± 0.12
8	0.69 ± 0.12	0.70 ± 0.12	0.76 ± 0.14	0.85 ± 0.15	0.75 ± 0.15
9	0.38 ± 0.07	0.38 ± 0.07	0.40 ± 0.09	0.51 ± 0.11	0.42 ± 0.10
10	0.74 ± 0.10	0.87 ± 0.16	0.89 ± 0.14	0.95 ± 0.16	0.86 ± 0.16
11	0.44 ± 0.08	0.46 ± 0.08	0.54 ± 0.11	0.54 ± 0.09	0.50 ± 0.10

### Myosignals and extracted muscle synergies

**Fig. 5:**
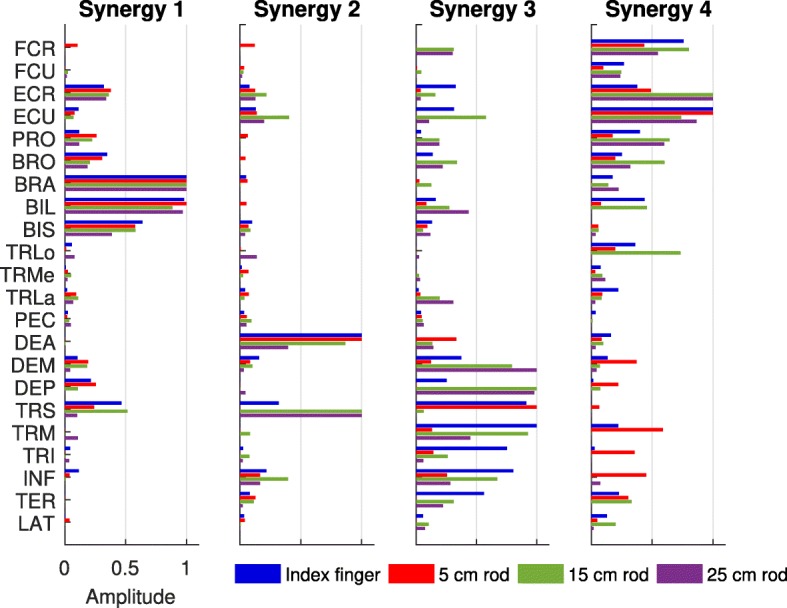
Example of an extracted set of muscle synergies for every end-effector, for one representative participant. The height of the bar indicates the proportional activation levels of a certain muscle in the synergy. Before being depicted, muscle synergies were matched across end-effectors based on their NDP

**Fig. 6:**
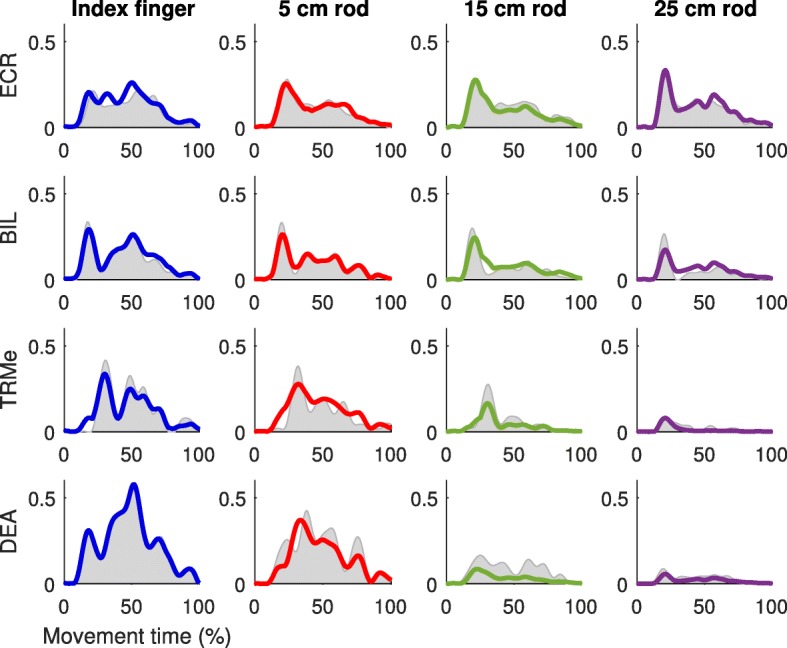
Example of reconstruction of myosignals of one representative participant. Myosignals depicted were measured from a center-out movement towards target 1. Grey areas represent the processed EMGs and colored lines the reconstruction by muscle synergy combinations

In about 1% of the signals, myosignals showed divergent patterns, probably due to the partial detachment of the electrode from the skin. These myosignals were removed from the analysis. For the remaining and approved myosignals, for each end-effector and for each participant, 3–5 muscle synergies—an example can be seen in Fig. 5—were able to explain 73.8–81.1% of data variance (index finger: 76.1 ± 4.1%; 5 cm rod: 76.6 ± 4.7%; 15 cm rod: 73.8 ± 5.2%; 25 cm rod: 81.1 ± 5.4%; as averaged across participants, also see Table 2). Importantly, across all participants, 6–8 muscle synergies extracted from the pooled dataset including myosignals from all end-effectors could equally well explain the observed myosignals per end-effector as the muscle synergies extracted separately from the myosignals of these end-effectors could. The values in explained variances showed that for every end-effector, it was possible to reconstruct a considerable part of the observed myosignals with a limited number of muscle synergies (Fig. 6). Importantly, different myosignals were used when point-to-point movements were made with different end-effectors (Fig. 6). For instance, for a representative participant, the activity of the long part of the biceps brachii, medial part of the triceps, and anterior part of the deltoid declined, whereas the activity of the extensor carpi radialis slightly increased if longer rods were used (Fig. 6).

**Table 2:** Number of extracted muscle synergies with the amount of explained variance for each participant and end-effector

Participant	Index Finger	5 cm rod	15 cm rod	25 cm rod
1	3 / 80.7%	3 / 83.7%	4 / 81.6%	4 / 85.9%
2	4 / 80.5%	4 / 77.7%	3 / 73.2%	3 / 85.8%
3	4 / 75.2%	4 / 70.5%	3 / 69.5%	3 / 81.3%
4	4 / 76.6%	5 / 79.5%	5 / 79.5%	4 / 79.1%
5	4 / 76.9%	5 / 80.9%	4 / 75.0%	4 / 82.0%
6	4 / 78.1%	4 / 81.7%	4 / 73.3%	4 / 86.5%
7	4 / 72.0%	4 / 68.8%	4 / 67.1%	3 / 73.9%
8	3 / 66.4%	4 / 72.6%	4 / 68.0%	4 / 74.4%
9	4 / 77.1%	4 / 76.1%	4 / 75.4%	4 / 84.7%
10	4 / 78.4%	4 / 75.5%	4 / 68.4%	4 / 73.5%
11	4 / 74.9%	4 / 76.1%	4 / 80.6%	4 / 84.8%

No significant differences in number of muscle synergies across conditions (χ^2^ (3) = 4.20, *p* > 0.05)

### Assessment of the exploitation of muscle synergies

#### Comparison of the structure of individual muscle synergies extracted from separate end-effectors

Visual inspection of the similarity of structure of individual muscle synergies led to a first indication of a partial dissimilarity in muscle synergy structure across end-effectors. This indication was guided by the observation that the proportional activation levels between muscles within part of the individual muscle synergies (as indicated by the height of the bars in Fig. 5) were unequal across end-effectors. For instance, for the muscle synergies of a representative participant (Fig. 5), the contribution of shoulder and back muscles in muscle synergy 3 and wrist muscles in muscle synergy 4 varied across end-effectors. Notably, the structures of muscle synergies 1 and 2 of this particular participant were much more similar across end-effectors (Fig. 5).

**Fig. 7:**
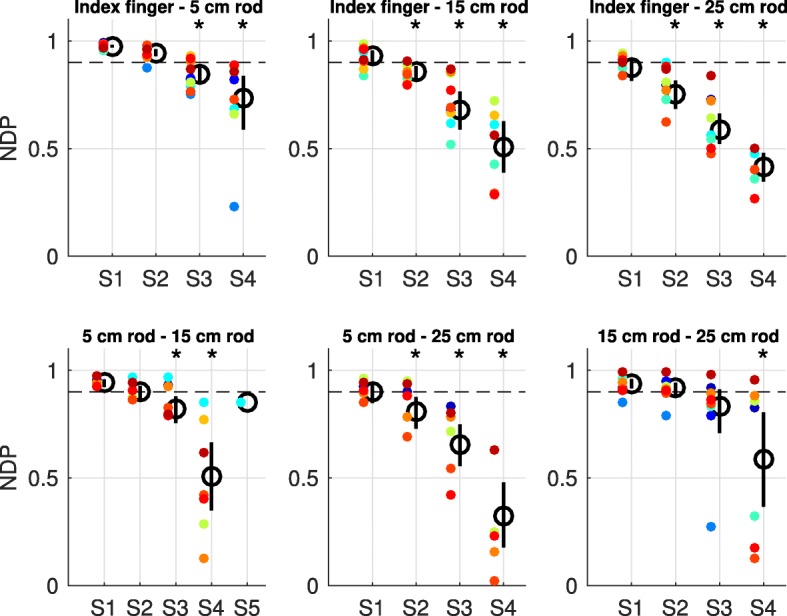
NDPs of the end-effector-pair-wise comparison of individual muscle synergies. NDPs were averaged across participants (means indicated by the black circles) and ranked from the best matching muscle synergies (S) to the least matching muscle synergies between two end-effectors. Each panel presents the results of the comparison of individual muscle synergies between two end-effectors. Vertical bars represent the 95% confidence interval determined with bootstrap statistics, whereas colored dots represent data of individual participants. Asterisks indicate mean NDPs that are significantly different from threshold (horizontal dashed line, significance determined with the 95% confidence interval)

The NDP analysis performed on muscle synergy pairs between two end-effectors confirmed the visual observation. This analysis showed that for every end-effector at least one muscle synergy differed in structure with the muscle synergies from another end-effector (as indicated by the 95% confidence interval, Fig. 7). Interestingly, the number of dissimilar muscle synergies between end-effectors gradually became larger as the absolute difference in length between these end-effectors was larger (Fig. 7). For instance, the total muscle synergy set extracted from the index finger exhibited two muscle synergies which structure was significantly dissimilar from the muscle synergies from the 5 cm rod and three muscle synergies which structure was significantly dissimilar from the muscle synergies from the 15 cm and 25 cm rods (Fig. 7). Notably, every end-effector-pair-wise comparison of muscle synergies also revealed muscle synergies that had a similar structure across end-effectors, indicating that these muscle synergies were exploited in movements with both end-effectors.

**Fig. 8:**
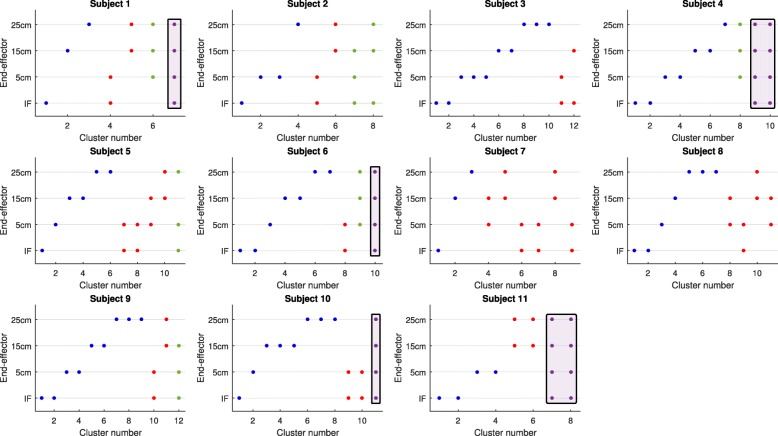
Distribution of the muscle synergies from the different end-effectors into the different clusters. Every panel presents the results of the clustering process for each individual participant. Before being depicted in the figure, clusters were ranked based on their size. Different colors indicate the size of the clusters: blue for one muscle synergy, red for two muscle synergies, green for three muscle synergies, and purple for four muscle synergies. Clusters with four members, i.e. indicating muscle synergies exploited across all end-effectors, are highlighted

The clustering of muscle synergies with a similar structure across all end-effectors showed that only half of the participants exhibited one or two muscle synergies that were exploited in every end-effector (Fig. 8; highlighted blocks indicate clusters with muscle synergies exploited across all end-effectors). Furthermore, for all participants, the majority of the clusters represented muscle synergies that were exploited in one end-effector or two end-effectors only. In addition, 7–12 clusters were needed to cluster all unique muscle synergies as observed in each participant (Fig. 8). This number of clusters was larger than the number of muscle synergies extracted from each end-effector (range 3–5), but smaller than the sum of number of muscle synergies across end-effectors (range 14–18), further confirming the partial dissimilarity in individual muscle synergy structure across all end-effectors.

#### Comparison of the explanatory ability of total muscle synergy sets extracted from separate end-effectors

**Fig. 9:**
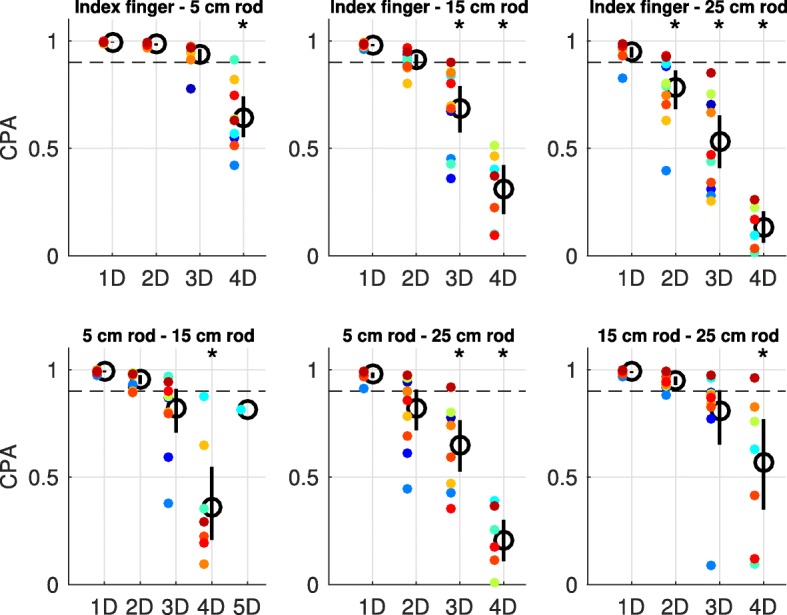
CPAs of the end-effector-pair-wise comparison of total muscle synergy sets. CPAs were averaged across participants (means indicated by the black circles) and ranked from the best matching dimensions (D) to the least matching dimensions between two end-effectors. Each panel presents the results of the comparison of subspaces spanned by the total muscle synergy sets of two end-effectors. Vertical bars represent the 95% confidence interval determined with bootstrap statistics, whereas colored dots represent data of individual participants. Asterisks indicate mean CPAs that are significantly different from threshold (horizontal dashed line, significance determined with the 95% confidence interval)

The comparison of subspaces spanned by the total muscle synergy sets from every end-effector showed that every subspace presented dimensions that were end-effector-specific (Fig. 9). Notably, every total muscle synergy set also shared dimensions of their subspace with the subspace from the total muscle synergy set from another end-effector (Fig. 9). Together, the comparison of subspaces showed that the total muscle synergy sets from the different end-effectors explained partially different subspaces in myosignal space. Interestingly, the total muscle synergy sets from the different end-effectors gradually spanned more distinct subspaces as the absolute difference in length of between end-effectors was larger (Fig. 9). For example, the total muscle synergy set extracted from the index finger shared a common 3D, 2D, and 1D subspace with the total muscle synergy set extracted from the 5 cm, 15 cm, and 25 cm rod, respectively (Fig. 9).

**Fig. 10:**
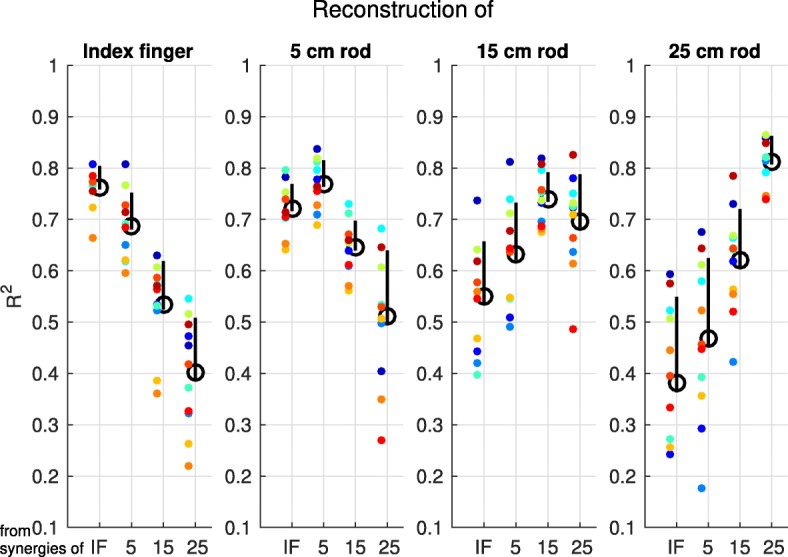
Explained variance of myosignals from the different end-effectors with the various total muscle synergy sets. Mean explained variance, indicated by the black circles, as averaged across participants, for the reconstruction of data from one end-effector using the set of muscle synergies extracted from the same or another end-effector. Upper vertical bars represent the standard deviation, lower vertical bars the standard error of the mean, and colored dots represent data of individual participants

The reconstruction of myosignals from one end-effector with the total muscle synergy set from another end-effector showed that the part of myosignal variance that a total muscle synergy set could explain decreased if this set had to explain myosignals from another end-effector as from which it was extracted (Fig. 10 [IF panel: F(3;30) = 126.21, *p* < 0.001, *η*^*2*^_*G*_ = 0.93; 5 cm rod panel: F(1.15;11.52) = 35.42, *p* < 0.001, *η*^*2*^_*G*_ = 0.78; 15 cm rod panel: F(1.25;12.49) = 16.88, *p* < 0.001, *η*^*2*^_*G*_ = 0.63; 25 cm rod panel: F(1.20;12.01) = 60.87, *p* < 0.001, *η*^*2*^_*G*_ = 0.86]). Per panel presented in Fig. 10, these ANOVA effects were further examined using pair-wise post-hoc t-tests, in which the portion variance of myosignals explained by ‘own’ total muscle synergy sets—e.g. index finger myosignals explained with index finger muscle synergies—was compared with the portion variance of myosignals explained with ‘other’ total muscle synergy sets—e.g. index finger myosignals explained with 5 cm rod muscle synergies. All except one (i.e. the explanation of 15 cm rod myosignals with 25 cm rod muscle synergies, *p* = 0.062) of these comparisons showed that the portion variance of myosignals as explained with other total muscle synergy sets was significantly lower as compared to the portion variance of myosignals explained with the own total muscle synergy set. Interestingly, and in line with the results presented above, this decreased ability to explain myosignals from other end-effectors was larger if the absolute difference in the length of the end-effectors from the extracted muscle synergies and to-be-explained myosignals was larger (Fig. 10).

#### Evaluation of the contribution of muscle synergies extracted from the pooled dataset

**Fig. 11:**
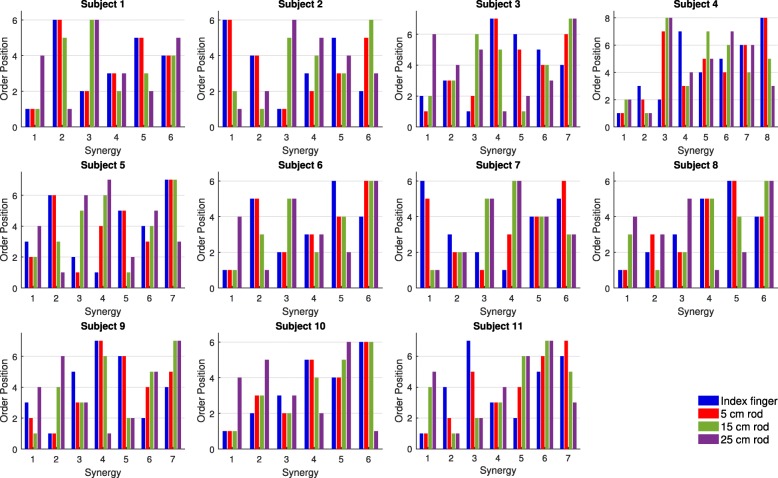
Position in contribution order for every end-effector for individual muscle synergies from the pooled dataset. Orders were ranked from 1 (most contributing) to 6–8 (least contributing)

**Fig. 12:**
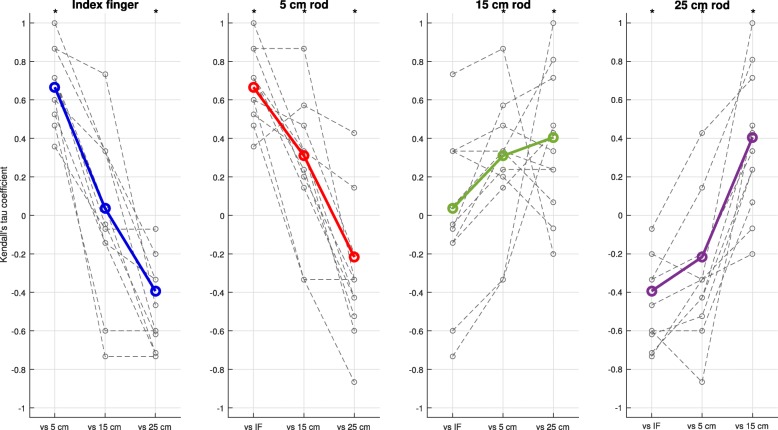
Kendall’s tau coefficients between the order of muscle synergy contribution of the different end-effectors. Every panel represents the coefficients for the comparison of orders of one end-effector with the orders of all other end-effectors. Colored solid lines represent coefficients including all participants, grey dashed lines represent coefficients per participant. Asterisks indicate coefficients including all participants significantly different from zero

Last, across end-effectors, different orders of contribution to the explanation of myosignals were found for the muscle synergies as extracted from the pooled dataset (Fig. 11). For instance, for participant 1, muscle synergy 2 contributed least to explanation of myosignals observed in index finger and 5 cm rod movements but contributed most to the explanation of myosignals observed in 25 cm rod movements (Fig. 11). Similar effects were observed in other participants and for other muscle synergies (Fig. 11). The comparison of orders between end-effectors showed that the difference between the order of muscle synergy contribution gradually became larger as the absolute difference in length between two end-effectors was larger (Fig. 12). For instance, in the left panel of Fig. 12, the order of muscle synergy contribution was similar between the index finger and 5 cm rod condition (positive correlation), whereas the order of muscle synergy contribution gradually changed to a reversed order between the index finger and 25 cm rod condition (negative correlation). Similar directions of effect—i.e. larger differences between orders of muscle synergy contribution as absolute differences in end-effector lengths were larger—were also found in the other panels, indicating the reversal in the contribution of muscle synergies extracted from the pooled dataset across end-effectors.

## Discussion

The successful improvement of the intuitive control of myoelectric AT for upper extremities with the fixed muscle synergy approach requires that a limited number of muscle synergies is systematically exploited when muscles are used differently to perform a certain task, as in myoelectric AT. To assess whether this requirement is met, the present study examined the systematic exploitation of muscle synergies when multidirectional point-to-point movements had to be performed with end-effectors of different lengths. Results showed that 3–5 muscle synergies could explain a substantial part of the myosignals *within* every end-effector. Furthermore, the same part of myosignals could be explained with 6–8 muscle synergies extracted from a pooled dataset including all myosignals *across* end-effectors. These results showed that the requirement that a limited number of muscle synergies was systematically exploited to account for myosignals when muscles were used differently during the performance of the same task—i.e. using end-effectors of different lengths—was met. Hence, these results indicated that there is a potential for the fixed muscle synergy approach to improve the intuitive control of myoelectric AT for upper extremities for the set of movements within the specific task as examined in the present study.

In line with previous studies examining the presence of muscle synergies in upper extremity myosignals [38–50], the present findings showed that within and across end-effectors muscle synergies could explain a considerable part of the variation in observed myosignals. Subsequent analyses showed in a three-step evaluation that the exploitation of muscle synergies gradually changed across end-effectors. First, the structure of individual muscle synergies—i.e. the proportional activation levels of muscles within a muscle synergy—gradually became more dissimilar as the difference in end-effector length was larger. Second, total muscle synergy sets from two different end-effectors gradually spanned more distinct subspaces as end-effectors differed more in length. Moreover, the ability of a total muscle synergy set from one end-effector to explain myosignals from another end-effector gradually decreased as a function of the difference in length of the end-effectors. Third, the order of contribution of muscle synergies extracted from the pooled dataset gradually reversed across end-effectors. This change in the exploitation of muscle synergies across end-effectors is in agreement with studies that observed the use of different upper extremity muscle synergies across conditions [41, 44, 48–50]. Such a changing exploitation of muscle synergies across conditions could indicate that the total pool of fixed muscle synergies represented at the spinal cord is larger than the set of fixed muscle synergies that is used in every condition [48, 74]. It is suggested that from this total pool, the appropriate set of fixed muscle synergies for a certain condition can be selected to produce the required myosignals, which is the core assumption of the fixed muscle synergy approach. The question relevant for the control of myoelectric AT is how muscle synergies can help in improving the intuitive control of this AT.

### On the implementation of muscle synergies to control myoelectric AT for upper extremities

Depending on whether muscle synergies were extracted from separate end-effectors or from the pooled dataset, we found different numbers of muscle synergies that could explain the myosignals. Therefore, we see two different options for the implementation of the idea of fixed muscle synergies to improve the intuitive control of myoelectric AT for upper extremities. First, within a limited set of movements—such as made within every end-effector in our case—3-5 muscle synergies could control a myoelectric assistive device that can be used for this limited set of movements. Given the small number of control variables—e.g. the 3–5 muscle synergies—such a device will have a rather direct relation between the activation of these muscle synergies and the restricted set of movements. Second, when interested to control a device for a broader set of movements within a specific task, which allows for more flexibility, one could use the expanded set of 6–8 muscle synergies. Given the reversal of the contribution of these muscle synergies to the observed movements across end-effectors, the activation of these 6–8 muscle synergies needs to be scaled based on the movement that is intended to be produced—i.e. one should select the muscle synergies that contribute most to the intended movement. Such a control scheme resembles the idea presented above of selecting a fixed muscle synergy from a total pool represented at the spinal cord.

Both implementation options presented here fit with the concept of pattern recognition control (cf. [2, 75, 76]). In pattern recognition control, a software algorithm detects combinations of features in a set of myosignals and classifies them into one of the multiple possible actions of the actuators in the AT. To control such pattern recognition based assistive devices, the user has to produce a certain movement such that the device can recognize, and subsequently produce, the desired action that is linked to this specific movement. Given the intuitive relation between pattern recognition control and the fixed muscle synergy approach (cf. [77]), it might be that the intuitive control of myoelectric AT can benefit from the implementation of muscle synergies in a pattern recognition control scheme. For instance, because the opening and closing of the hand during healthy grabbing actions can be correlated with different phases of the transportation of the arm through space [78], the opening and closing of a prosthetic hand might be linkable to the patterns in myosignals—i.e. muscle synergies—during this transportation. Such a link between the actions performed with myoelectric AT and the muscle synergies that are exploited during the healthy production of these actions might improve the intuitive control of myoelectric AT for upper extremities.

Previous work on the implementation of muscle synergies to control of myoelectric AT has shown that muscle synergies are suitable for force estimation, and thus proportional control of myoelectric AT for upper extremities [79, 80]. However, as muscle synergies do not exceed the performance of myosignals of individual muscles in classifying movements [80] or reconstructing the movements of the end-effector [81, 82], the benefit of using muscle synergies as compared to currently used myosignals to control myoelectric AT is unclear. Furthermore, in both myosignal and muscle synergy control, considerable errors in classifying or reconstruction performance are observed [80–82]. For instance, both myosignals of individual muscles and muscle synergies can be used equally well to online control the movement of a virtual object to reach a target location [82], yet both types of control yield for considerable errors in task performance. These results suggest that muscle synergies, and maybe also myosignals, lack important information that is used in the production of behavior—information that is thus important for the intuitive control of myoelectric AT (cf. [31, 83–85]). Therefore, we recommend that future research should examine whether the actual implementation of muscle synergies is beneficial for the control of myoelectric AT for upper extremities.

Furthermore, both implementation options presented here ground the potential of muscle synergies to improve the intuitive control of myoelectric AT for upper extremities on the specific set of movements within a task that these muscle synergies can explain. Therefore, it remains the question whether this potential can be generalized to a broader set of movements and tasks. The set of movements examined in the present task—i.e. movements with different end-effectors—could be captured with a relatively limited number of 6–8 muscle synergies. However, it is possible that—especially given the relatively large change in muscle synergies within the relatively small set of examined movements across end-effectors—more muscle synergies are needed when the set of movements is expanded to a wide range of tasks. If indeed increasingly more muscle synergies are needed to account for movements as produced in an increasing number of tasks, it might be debatable i) whether all these muscle synergies represent the use of fixed muscle synergies from a pool in the spinal cord, and ii) whether the idea of fixed muscle synergies is an attractive approach to implement in the control scheme of myoelectric AT that is ought to be used for a large range of tasks.

### Does the presence of muscle synergies originate from the activation of neural networks?

Another issue can be raised concerning the interpretation that muscle synergies as found in myosignals originate from the activation of fixed neural networks. In their studies on a cadaver arm and hand, Kutch & Valero-Cuevas [86] suggested that the presence of low-dimensional patterns in myosignals—i.e. muscle synergies—does not necessarily imply the activation of a fixed neural circuity—i.e. *fixed* muscle synergies. In this study, myosignals were estimated via a neuromechanical model based on i) measured forces acting on the cadaver muscles after an external perturbation of the end-effector, and ii) externally applied forces on the cadaver muscles to produce isometric forces in various directions at the end-effector. Subsequently, a principal component analysis showed a low-dimensional pattern in the estimated myosignals. Thus, besides the possibility of originating from neural circuitry, the presence of low-dimensional patterns in myosignals can also emerge from biomechanical—e.g. muscles that resist lengthening—and task constraints—e.g. direction of isometric force produced at the end-effector [86].

This conceptual discussion on whether muscle synergies as observed in myosignals originate from the activation of neural circuitry or emerge from biomechanical and task constraints is relevant for the applicability of muscle synergies to improve the intuitive control of myoelectric AT. When applying muscle synergies to the control of myoelectric AT, one needs to take into account that biomechanical and task constraints change when myoelectric AT is used in different situations. Given that these changing constraints affect the activation of muscles—for instance, muscles need to be activated differently depending on limb posture to produce the same limb motion [87] and muscles perform different roles in different contexts [88]—it is possible that also muscle synergies as observed in myosignals change on basis of these changing constraints. In fact, the gradual change in the exploitation of muscle synergies as found in the present study may have emerged from the subtle differences in task constraints as induced by the variation in end-effector length. Likewise, findings of the exploitation of similar upper extremity muscle synergies across conditions [38, 39, 43, 45] could have been the result of similarities in task constraints across these conditions. For instance, the visuomotor adaption paradigm as presented in Gentner et al. [45] required participants to maintain end-effector movements in the same plane of motion across conditions. These similarities in task constraints could have resulted in similar arm movements across conditions, hence explaining the findings of similar muscle synergies. If i) the majority of muscle synergies indeed emerges from biomechanical and task constraints instead of from the activation of fixed neural circuitry, and ii) observed muscle synergies change with changing constraints, basing the control scheme of myoelectric AT on muscle synergies that change with changing constraints might not a fruitful approach to pursue when aiming to improve the intuitive control of myoelectric AT.

Thus, following the discussion as presented in the last two paragraphs, the answer to the question of what the role of fixed muscle synergies is in the production of myosignals is not straightforward (cf. [31, 81, 85, 89]). Given that the majority of participants exhibited one or two muscle synergies that were shared across at least three end-effectors, it seems reasonable that some of the observed muscle synergies can be captured in fixed neural networks. However, based on the variation in the exploitation of the majority of muscle synergies within the relatively limited set of movements as examined in the present study, it is to be examined whether the neuromotor system solely uses an organization of fixed muscle synergies to produce myosignals.

### Alternatives for an intuitive control scheme for myoelectric AT

In the remainder of this discussion, we address two possible alternatives for designing an intuitive interface between user and device. First, we think that, as mentioned in the introduction, the intuitive control of myoelectric AT will benefit most from the connection of its control to principles that are already used by the neuromotor system (cf. [28–31]). In trying to connect neuromotor control principles to the control of myoelectric AT, it is important to consider that the environment in which neuromotor actions are conducted provides for perceptual information that is relevant for the control of these actions. Thus, considering the closing of the perception-action loop by including perceptual information in the technology—of which the lack also is assigned as a primary concern among myoelectric AT users [17–21]—might be a vital route in the improvement of the intuitive control of myoelectric AT. In line with this idea, it has been shown that providing feedback has beneficial effects on prosthesis control [90–93], see for reviews [94, 95]. Therefore, we underline the importance of future research towards *sensori*motor control principles and their link to the intuitive control of myoelectric AT for upper extremities.

Second, it is suggested that the displacement of control from muscles to neurons might help for the improvement of the intuitive control of AT. Over the last years, several routes in this endeavor have already been taken, e.g. redirecting remaining parts of nerves to remaining muscles [75, 96–98], and using discharges of individual spinal motoneurons [99–101] and of neurons in the motor cortex [102–104] to control the AT. However, to apply this technology in a general context—as an ideal assistive device ought to be designed—present technology requires that a systematic pattern can be recognized in the observed neural activity. Since we found variability in myosignal patterns—i.e. muscle synergies that varied across end-effectors—it is to be expected that also patterns in this neural activity will vary across conditions. Therefore, the same issues as raised above on the extent to which muscle synergies can be used in a general way to control myoelectric AT apply on the use of neural drive to control this AT. Following this rationale, arguably the design of the present neural approach is left with technology that might only be applied in a task- or condition-specific sense.

### Limitations

Although the present study examined the potential of fixed muscle synergies to improve patient’s intuitive control of myoelectric AT for upper extremities, such AT, nor patients, were included in the experiment. Nevertheless, we think that the used experimental set-up related well enough with the use of myoelectric AT, as the manipulation of end-effectors induced the different use of muscles for the task, as is required during the control of myoelectric AT. Furthermore, by including only healthy subjects into the protocol, the present study could examine the systematic exploitation of muscle synergies in the absence of confounding factors resulting from the actual use of myoelectric AT—such as its alternated feedback system and influence on dynamics of task performance. Thus, the present study offered an entrance to gain knowledge purely about the potential of the fixed muscle synergy approach to improve the intuitive control of myoelectric AT for upper extremities.

Furthermore, the relatively small sample size could be a limitation of the present study. Yet, our sample size falls in the range of number of participants used in other studies examining muscle synergies. Moreover, the direction of the effects was systematic across participants. Therefore, we do not expect differences in the results as reported in the present study if more participants would have been included.

## Conclusion

The present study demonstrated that a limited number of muscle synergies was systematically exploited during the production of myosignals in point-to-point movements with different end-effectors. This result indicated a potential for employing the fixed muscle synergy approach to improve the intuitive control of myoelectric AT for upper extremities for the set of movements within the examined task. It remains the question whether this potential can be extended to a larger range of movements and tasks. Future research should be aimed at examining the fixed character of muscle synergies as well as the generalization of the potential of the fixed muscle synergy approach to improve the intuitive control of myoelectric AT for upper extremities. This examination will be important to improve the intuitive control of assistive devices and, in broader terms, rehabilitation practice.

## Abbreviations

AT: Assistive technology
BIL: Long head of the biceps brachii
BIS: Short head of the biceps brachii
BRA: Brachialis
BRO: Brachioradialis
CPA: Cosine of principle angles
DEA: Anterior part of the deltoid
DEM: Middle part of the deltoid
DEP: Posterior part of the deltoid
ECR: Extensor carpi radialis
ECU: Extensor carpi ulnaris
EMG: Electromyography
FCR: Flexor carpi radialis
FCU: Flexor carpi ulnaris
INF: Infraspinatus
LAT: Latissimus dorsi
LED: Light-emitting diode
NDP: Normalized dot product
PEC: Pectoralis major
PRO: Pronator teres
R^2^: Explained variance
SSE: Sum of squared errors
SST: Sum of squared residuals
TER: Teres major
TRI: Inferior part of the trapezius
TRLa: Lateral head of the triceps brachii
TRLo: Long head of the triceps brachii
TRM: Medial part of the trapezius
TRMe: Medial head of the triceps brachii
TRS: Superior part of the trapezius
